# NF-κB RelB suppresses the inflammatory gene expression programs of dendritic cells by competing with RelA for binding to target gene promoters

**DOI:** 10.1038/s41421-024-00767-9

**Published:** 2025-02-11

**Authors:** Héctor I. Navarro, Allison E. Daly, Benancio Rodriguez, Sunny Wu, Kim A. Ngo, Anna Fraser, Allison Schiffman, Yi Liu, Stephen T. Smale, Jennifer J. Chia, Alexander Hoffmann

**Affiliations:** 1 Department of Microbiology, Immunology, and Molecular Genetics, University of California, Los Angeles, CA USA; 2 Molecular Biology Institute, University of California, Los Angeles, CA USA; 3 Institute for Quantitative and Computational Biosciences, University of California, Los Angeles, CA USA; 4 Jonsson Comprehensive Cancer Center, University of California, Los Angeles, CA USA; 5 Broad Stem Cell Research Center, University of California, Los Angeles, CA USA; 6 Department of Pathology and Laboratory Medicine, University of California, Los Angeles, CA USA; 7 Present Address: DeepKinase Biotechnologies Ltd., Beijing, China

**Keywords:** Innate immunity, Transcription, Cell signalling

## Abstract

A group of autoinflammatory disorders termed relopathies arise as a consequence of NF-κB dysregulation. Genetic loss of the NF-κB subunit RelB in humans and mice leads to autoimmunity and lethal multi-organ inflammatory pathology. Our recent study showed that this inflammatory pathology is independent of type I interferon signaling, and further identified dysregulation of a set of pro-inflammatory NF-κB target genes. However, it remains unknown how the loss of RelB leads to the dysregulation of these NF-κB motif-containing pro-inflammatory genes. Here, we report epigenome profiling studies revealing that RelB is associated with pro-inflammatory genes in dendritic cells. While these genes recruit RelA binding upon exposure to a maturation stimulus, we observed substantially more RelA recruitment in the absence of RelB. For these genes, we found that elevated RelA recruitment is correlated with elevated gene expression. To test whether RelB may compete with RelA for binding to NF-κB-regulated gene promoters via competition for κB sites, we generated a new mouse strain (*RelB*^*DB/DB*^) that harbors targeted point mutations in the RelB DNA binding domain that eliminates high-affinity DNA binding. We found that this targeted mutation in the RelB DNA binding domain is sufficient to drive multi-organ inflammatory pathology. These results provide insights into the biological mechanism of RelB as a suppressor of pro-inflammatory gene expression and autoimmune pathology.

## Introduction

Current studies estimate that there are ~150 life-long autoimmune diseases that are characterized by dysregulation of the adaptive immune system with no known cures^1^, affecting ~5%–8% of the world population^2^. There is also a growing list of over 40 genetically encoded autoinflammatory diseases characterized by the dysregulation of innate immune responses^3^. One of the most common immune response pathways associated with autoimmune and autoinflammatory diseases is NF-κB^4–8^. NF-κB plays a central role in inducing the expression of genes involved in cell survival, differentiation, and inflammation^9–14^. The NF-κB family is comprised of multiple hetero- and homo-dimeric transcription factors that are made up of the subunits RelA, cRel, RelB, p52, or p50^15,16^. Genetic loss of the RelB subunit in mice results in multi-organ inflammatory pathology characterized by several phenotypic lesions, including (1) mixed inflammatory cell infiltration in organs such as the lung and liver, (2) splenomegaly due to extramedullary hematopoiesis, and (3) thymic atrophy^17^. We previously identified a pronounced dysregulation of interferon (IFN)-stimulated gene (ISG) expression in fibroblasts obtained from a patient with combined immunodeficiency (CID) and autoimmunity due to a rare homozygous *RELB* mutation that results in complete loss of RelB protein^18–20^. This dysregulation in the IFN-stimulated gene program was also seen in dendritic cells (DCs) derived from *RelB*^*−*^^/^^*−*^ mice. Several studies have implicated the loss of RelB in DCs as a key driver of autoimmunity in *RelB*^*−*^^/^^*−*^ mice, demonstrating the sufficiency of either the absence or presence of RelB in DCs alone in promoting or suppressing the inflammatory pathology, respectively, in the lung, liver, spleen and thymus^21–23^. Transcriptomic profiling in DCs revealed large-scale upregulation of ISGs, raising the possibility that pathology arising from loss of RelB may be an interferonopathy, a group of inherited autoinflammatory diseases characterized by dysregulation of the IFN pathway^24–27^. However, genetic ablation of IFN signaling in *IFNAR*^−/−^*RelB*^*−*^^/^^*−*^ mice did not improve critical aspects of the *RelB*^−/−^ pathology^20^. Instead, further analysis of DCs derived from *IFNAR*^*−*^^/^^*−*^*RelB*^*−*^^/^^*−*^ mice revealed a subset of dysregulated NF-κB motif-containing pro-inflammatory genes that are likely drivers of *RelB*^*−*^^/^^*−*^ pathology, and suggested that it is in fact a relopathy (an NF-κB/Rel-associated autoinflammatory disease)^28–30^. The observations that NF-κB proinflammatory genes were hyper-expressed in the setting of RelB loss in patient-derived fibroblasts, and that NF-κB signaling was dysregulated in a separate study of patients harboring a different *RELB* mutation leading to reduced RelB protein^31^, together call for further investigation of how the loss of RelB activity leads to the dysregulation of NF-κB motif-containing pro-inflammatory genes.

RelB is thought to both activate and repress transcription of metabolic and inflammatory genes, while RelA is a potent transcriptional activator that induces inflammatory gene expression^32–34^. In the absence of stimulation, RelB is localized to both the nucleus and cytoplasm. RelB can form dimers with RelA that are non-functional in binding DNA or IκBs^35^, presumably because they have an unusually intertwined quaternary structure^36^. In the cytoplasm, RelB can also stabilize p100/p105 interactions that sequester RelA in a non-productive complex^37–42^. In the nucleus, RelB can repress gene expression activity by competing with RelA for binding κB sites^43^ and potentially facilitate repressive chromatin modifications via histone deacetylase and histone methyltransferase activities^44,45^. RelB is also implicated in regulating DNA methylation via the Daxx protein^46^ and in increasing IκBα stability^47^. However, it remains unclear which of these mechanisms is responsible for mediating autoimmunity in RelB-deficient patients and mice^18,19^. Given the direct role of RelB loss in DCs in promoting autoimmune pathology, the role of DCs in other autoimmune diseases^21–23,48–51^, and the high expression of RelB in healthy mouse and human DCs^52–54^, we sought here to identify the mechanism by which RelB represses inflammatory gene expression in DCs. We found that RelB is in fact associated with pro-inflammatory genes in DCs prior to maturation stimulation. Upon stimulation, RelA is recruited to NF-κB response genes, and this recruitment is potentiated in *RelB*^*−*^^/^^*−*^ DCs at locations that were bound by RelB prior to stimulation. To test the role of RelB DNA binding in functional DC responses, we then generated a RelB DNA binding mutant mouse strain (*RelB*^*DB/DB*^), in which RelB lacks three amino acid side chains that make specific contacts with κB site nucleotides. Phenotypic analysis of these mice and genome-wide RNA-seq and ChIP-seq analyses performed in DCs derived from them, showed that the RelB DNA binding mutant is defective in suppressing the inflammatory pathology. Our results support a model in which RelB competes with RelA for κB sites, and thus RelB functions to dampen the expression of immune response genes in DCs and reduces the risk for auto-inflammatory and autoimmune disease.

## Results

### Characterization of IFN-independent multi-organ inflammation in *RelB*^*−*^^/^^*−*^ mice

**Fig. 1:**
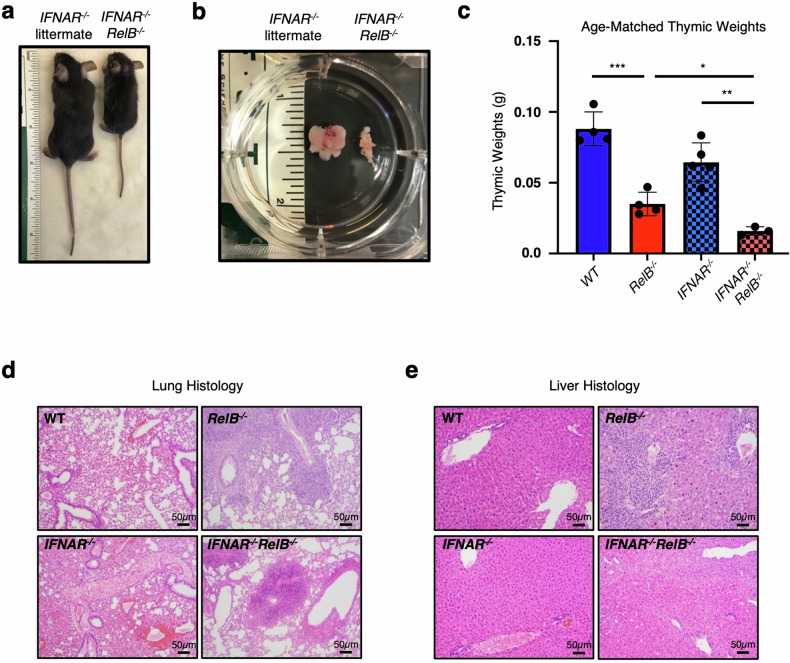
Characterization of IFN-independent multi-organ inflammation in *RelB*^*−*^^/^^*−*^ mice. **a** Representative image of *IFNAR*^*−*^^/^^*−*^*RelB*^*−*^^/^^*−*^ mouse (right) and *IFNAR*^*−*^^/^^*−*^ littermate (left) at 4 weeks of age; ruler for scale. **b** Representative image of thymi from *IFNAR*^*−*^^/^^*−*^*RelB*^*−*^^/^^*−*^ mouse (right) and *IFNAR*^*−*^^/^^*−*^ littermate (left). **c** Thymic weights from age-matched WT (dark blue), *RelB*^*−*^^/^^*−*^ (dark red), *IFNAR*^*−*^^/^^*−*^ (light blue, checkered), *IFNAR*^*−*^^/^^*−*^*RelB*^*−*^^/^^*−*^ (light red, checkered) mice. ***P < 0.001, **P < 0.01, *P < 0.05; error bars indicate SD. Statistical analysis was done using an unpaired two-tailed Student’s *t*-test. **d**, **e** Representative images of hematoxylin and eosin (H&E)-stained sections of WT, *RelB*^*−*^^/^^*−*^, *IFNAR*^*−*^^/^^*−*^, and *IFNAR*^*−*^^/^^*−*^*RelB*^*−*^^/^^*−*^ mouse lungs (**d**) and livers (**e**). *n* = 3–4.

Loss of RelB in mice results in multi-organ inflammatory pathology, including mixed lymphocytic and granulocytic infiltrates in the lung and liver, splenomegaly due to increased extramedullary hematopoiesis, and thymic atrophy^17^. Transcriptomic studies performed in both patient fibroblasts harboring a loss-of-function *RELB* mutation and DCs from *RelB*^*−*^^/^^*−*^ mice revealed a pronounced dysregulation of ISG expression^20^, raising the possibility that RelB deficiency may be characterized as an interferonopathy. However, we recently showed that genetic ablation of type I IFN signaling in an *IFNAR*^*−*^^/^^*−*^*RelB*^*−*^^/^^*−*^ compound knockout mouse strain did not improve critical aspects of RelB-deficient pathology, including unchanged splenomegaly, runted growth, and elevated serum cytokine levels^20^. These surprising findings have prompted us to examine other aspects of RelB deficiency including thymic atrophy and visceral inflammation that have been previously reported^17,22,23^. As previously reported, *IFNAR*^*−*^^/^^*−*^*RelB*^*−*^^/^^*−*^ mice showed runted growth compared to *IFNAR*^*−*^^/^^*−*^ controls (Fig. 1a), and displayed splenomegaly^20^. Thymi from *IFNAR*^*−*^^/^^*−*^*RelB*^*−*^^/^^*−*^ mice were smaller in size (Fig. 1b) and weight (Fig. 1c) than *IFNAR*^*−*^^/^^*−*^ controls, indicating thymic atrophy similar to that previously described in *RelB*^*−*^^/^^*−*^ mice^17^. Histologic sections of the lung and liver appeared unremarkable in wild-type (WT) and *IFNAR*^*−*^^/^^*−*^ mice; in contrast, there was moderate to marked mixed lymphocytic and granulocytic inflammation arranged in diffuse and perivascular distributions in the lungs and livers of *IFNAR*^*−*^^/^^*−*^*RelB*^*−*^^/^^*−*^ mice (Fig. 1d, e), again similar to the previously described findings for *RelB*^*−*^^/^^*−*^ mice^17^. Previous studies have shown that RelB deficiency limited to DCs is sufficient to drive inflammatory pathology in the liver, spleen, lung, and thymus of *RelB*^*−*^^/^^*−*^ mice^21–23^. While IFN signaling was shown to be dysregulated in DCs derived from *RelB*^*−*^^/^^*−*^ mice^20^, the phenotypic and histopathologic features of *IFNAR*^*−*^^/^^*−*^*RelB*^*−*^^/^^*−*^ mice provide further evidence that the pathology arising upon RelB deficiency is largely driven independently of IFN signaling.

### Loss of RelB leads to elevated RelA binding at genomic RelB-bound κB sites

**Fig. 2:**
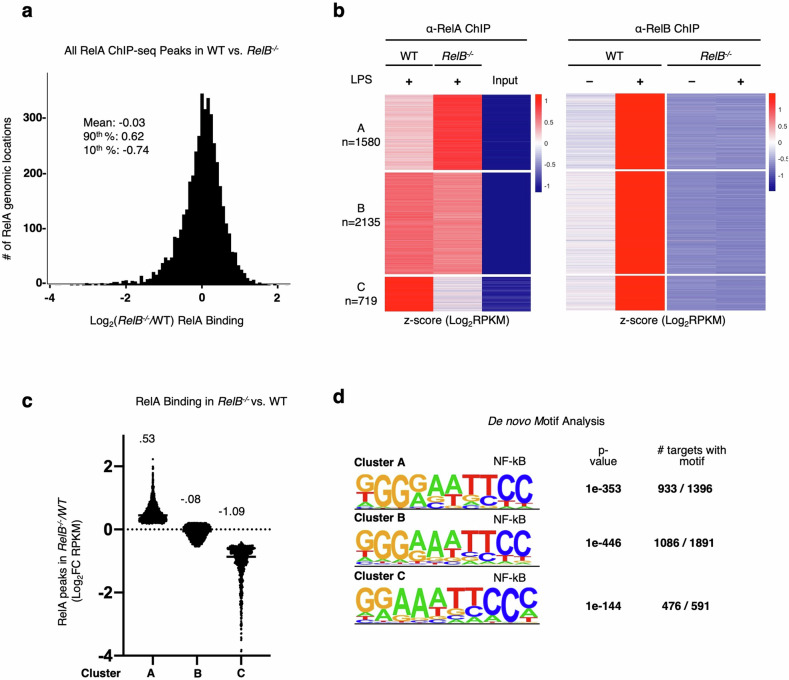
Loss of RelB leads to elevated RelA binding to a subset of genomic κB sites. **a** Histogram of log_2_FC (*RelB*^−/−^/WT) in RelA binding (RPKM) for all ChIP peaks with RPKM > 10. Descriptive statistics: mean, log_2_FC = −0.03; 90th percentile, log_2_FC = 0.62; 10th percentile, log_2_FC = −0.74. **b** Heatmap of *z*-scored RelA- and RelB-ChIP peaks (RPKM) in all RelB-bound peaks in WT and *RelB*^−/−^ BMDCs. Peaks selected for all LPS (10 ng/mL)-induced RelB-ChIP binding events (log_2_FC > 1) at 1 h LPS stimulation time point in WT BMDCs, RPKM > 10 in any condition. Heatmaps for RelA- and RelB-ChIP-seq data are ordered by RelA k-means clustering and separately *z*-scored. **c** Scatter plot showing the means of log_2_FC (*RelB*^−/−^/WT) in RelA binding for clusters A–C from **b**. **d** Top de novo motif analysis results for genomic regions from clusters A–C from **b**.

Considering these findings, we focused our attention on the previously identified NF-κB-driven pro-inflammatory gene expression that remained dysregulated in bone marrow-derived dendritic cells (BMDCs) derived from *IFNAR*^*−*^^/^^*−*^*RelB*^*−*^^/^^*−*^ mice^20^ and was also dysregulated in patient-derived fibroblasts from RelB-null patients^20^. We asked whether RelB and RelA may compete for binding to the same κB sites, as RelB-containing heterodimers have shown DNA binding specificities similar to RelA heterodimers^55^^,56^. We approached this question by first examining whether the loss of RelB results in altered RelA binding at NF-κB motifs that would normally be bound by RelB in WT conditions. To answer this, we generated BMDCs from both *RelB*^*−*^^/^^*−*^ and WT mice and performed ChIP-seq for the NF-κB subunits RelA and RelB. We first analyzed the distribution of the relative binding signal of all RelA binding sites between *RelB*^−/−^ and WT BMDCs and found that they were normally distributed and centered at a 1:1 ratio. Binding sites at the 90th percentile displayed a log_2_fold change (FC) of 0.62 in binding between *RelB*^−/−^ and WT BMDCs, whereas binding events in the bottom 10% displayed a log_2_FC of –0.74 (Fig. 2a; Supplementary Table S1). To assess whether sequences bound by RelB in WT BMDCs display altered RelA binding in *RelB*^−/−^ BMDCs, we selected RelB binding events induced > 2-fold by LPS stimulation (10 ng/mL) in WT BMDCs and analyzed the subsequent changes in RelA binding at these locations in *RelB*^−/−^ BMDCs. K-means clustering recognized three predominant RelA binding patterns at these sites (Fig. 2b). Cluster A (*n* = 1580) contained binding events that have elevated binding by RelA in *RelB*^−/−^ BMDCs with an average of log_2_FC = 0.53 between *RelB*^−/−^ and WT BMDCs (Fig. 2b, c; Supplementary Table S1). Cluster B (*n* = 2135) contained RelA binding events that were unchanged in *RelB*^−/−^ BMDCs with an average of log_2_FC = −0.08 between *RelB*^−/−^ and WT BMDCs (Fig. 2b, c; Supplementary Table S1), and binding events in cluster C (*n* = 719) displayed a reduction in RelA binding with an average of log_2_FC = −1.09 between *RelB*^−/−^ and WT BMDCs (Fig. 2b, c; Supplementary Table S1). Furthermore, motif analysis of sequences within these clusters confirmed the specificity of these binding events to RelA and RelB with ~60%–80% of peaks within each cluster containing NF-κB binding elements (Fig. 2d). These data reveal that while the majority of RelA DNA binding events are unchanged in the absence of RelB, the loss of RelB affects binding of RelA to a portion of NF-κB binding sites that are also bound by RelB in a WT setting. Gene ontology (GO) analysis of genes annotated to genomic sites in each of the clusters revealed “NF-kappaB signal transduction” (Cluster A, *P*-value = 7.75E–03; Cluster B, *P*-value = 2.25E–02), “innate immune response” (Cluster A, *P*-value = 5.61E–03; Cluster B, *P*-value = 1.84E–02) and “inflammatory response” (Cluster A, *P*-value = 3.76E–02; Cluster B, *P*-value = 4.88E–04) terms enriched in genes from clusters A and B; these terms were absent from genes in cluster C. Instead, the top terms in cluster C were “heart morphogenesis” (*P*-value = 4.95E–02), “cell junction disassembly” (*P*-value = 3.34E–02), and “NK cell-mediated cytotoxicity immune response” (*P*-value = 1.18E–02). These data suggest that RelA and RelB may compete throughout the genome for binding to NF-κB binding elements at or near genes with known inflammatory functions.

### Elevated RelA binding to promoter regions is correlated with elevated gene expression

**Fig. 3:**
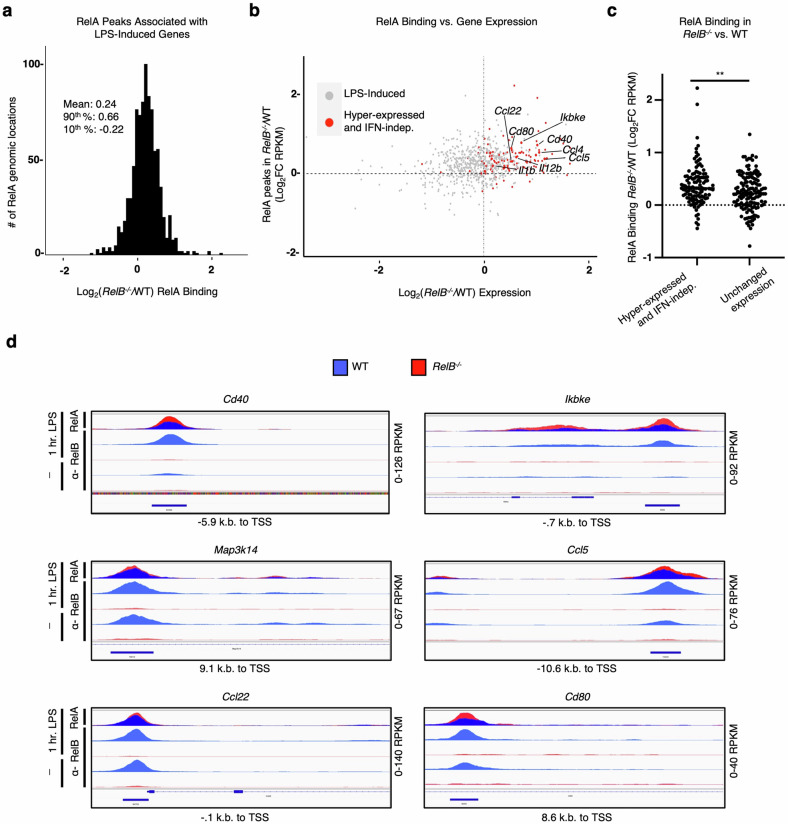
Elevated RelA binding to promoter regions is correlated with elevated gene expression. **a** Histogram of filtered highest FC RelA-ChIP peaks for all genes. Peaks were annotated to the nearest gene promoter region and filtered for the highest log_2_FC (*RelB*^−/−^/WT) in RelA binding (RPKM) for each individual gene; all peaks are RPKM > 10. Descriptive statistics: mean, log_2_FC = 0.24; 90th percentile, log_2_FC = 0.66; 10th percentile, log_2_FC = −0.22. **b** Scatter plot showing the log_2_FC (*RelB*^−/−^/WT) in RelA binding (RPKM) at 1 h LPS stimulation compared to all CpG-induced genes (Log_2_FC > 1) in WT or *RelB*^−/−^ BMDCs, 8 h gene expression time point shown. Previously characterized IFN-independent hyper-expressed genes in *RelB*^−/−^ are highlighted in red, and all other induced genes are colored gray. **c** Scatter plot comparing the log_2_FC (*RelB*^−/−^/WT) in RelA binding (RPKM) of IFN-independent hyper-expressed genes and genes with unchanged expression (−0.1 < log_2_FC < 0.1) in *RelB*^−/−^ relative to WT at 8 h CpG stimulation time point. **d** Representative genome browser tracks for ChIP peaks near promoter regions of TSS for IFN-independent hyper-expressed genes in *RelB*^−/−^. IGV tracks from WT BMDCs are represented in blue, and IGV tracks from *RelB*^−/−^ BMDCs are represented in red. *RelB*^−/−^ (red) and WT (blue) RelA-ChIP tracks (top row) are overlayed to provide a visual comparison.

We next sought to understand whether sites with elevated RelA binding in the absence of RelB are enriched at or near genes that are hyper-expressed in *RelB*^−/−^ BMDCs. We first annotated all RelA binding events to the nearest gene, resulting in some genes being annotated to several RelA binding events. Since we were specifically interested in RelA binding events that showed elevated binding as a consequence of the loss of RelB and associated with response to stimulation, we filtered our data to assign each gene to only the one RelA binding event that displayed the highest FC between *RelB*^−/−^ and WT BMDCs. After filtering, we found that the ratio between RelA binding events in *RelB*^−/−^ vs WT BMDCs was normally distributed and centered around 1.26. RelA binding events in the top 90th percentile displayed log_2_FC = 0.66 and the bottom 10th percentile had a log_2_FC = –0.22 (Fig. 3a; Supplementary Table S2). Using RNA-seq data sets from WT and *RelB*^−/−^ BMDCs, we then asked whether the pro-inflammatory genes previously reported to be hyper-expressed in *RelB*^−/−^ BMDCs contain NF-κB regulatory elements that resulted in elevated binding by RelA. To answer this, we selected genes induced > 2-fold upon TLR stimulation in either WT or *RelB*^−/−^ BMDCs, and then plotted the *RelB*^−/−^ hyper-expression phenotype (*x*-axis) against the elevated RelA binding phenotype (*y*-axis) and highlighted the previously reported IFN-independent NF-κB pro-inflammatory genes that were hyper-expressed in *RelB*^−/−^ BMDCs (red points) (Fig. 3b). We found that 98 of 117 (84%) of these genes fell within the elevated binding and hyper-expressed right upper quadrant (Fig. 3b). Further, loss of RelB resulted in a statistically significant increase (*P* < 0.01) in RelA binding at regulatory regions of genes that are hyper-expressed in an IFN-independent manner over genes whose expression is unchanged between *RelB*^−/−^ and WT BMDCs (Fig. 3c). We then compared the genome browser tracks of RelA binding events at or near the promoter regions of previously reported IFN-independent NF-κB pro-inflammatory genes that were hyper-expressed in *RelB*^−/−^ BMDCs^20^. While many RelA binding events were located at the promoter regions of these genes, the average distance between RelA binding events and their respective transcription start sites (TSSs) was −1352 bp (Supplementary Table S3). We observed that the loss of RelB resulted in elevated binding by RelA at or near the TSS of previously reported NF-κB pro-inflammatory genes including *Cd40*, *Ikbke*, *Map3k14*, *Ccl5*, *Ccl22*, and *Cd80* (Fig. 3d). These data provide evidence that RelA and RelB may compete for NF-κB binding sites, and that in a RelB-deficient context, hyper-expressed pro-inflammatory genes have elevated binding by RelA at or near their respective TSSs.

### A targeted mutant of the RelB DNA binding domain also shows autoimmune pathology

**Fig. 4:**
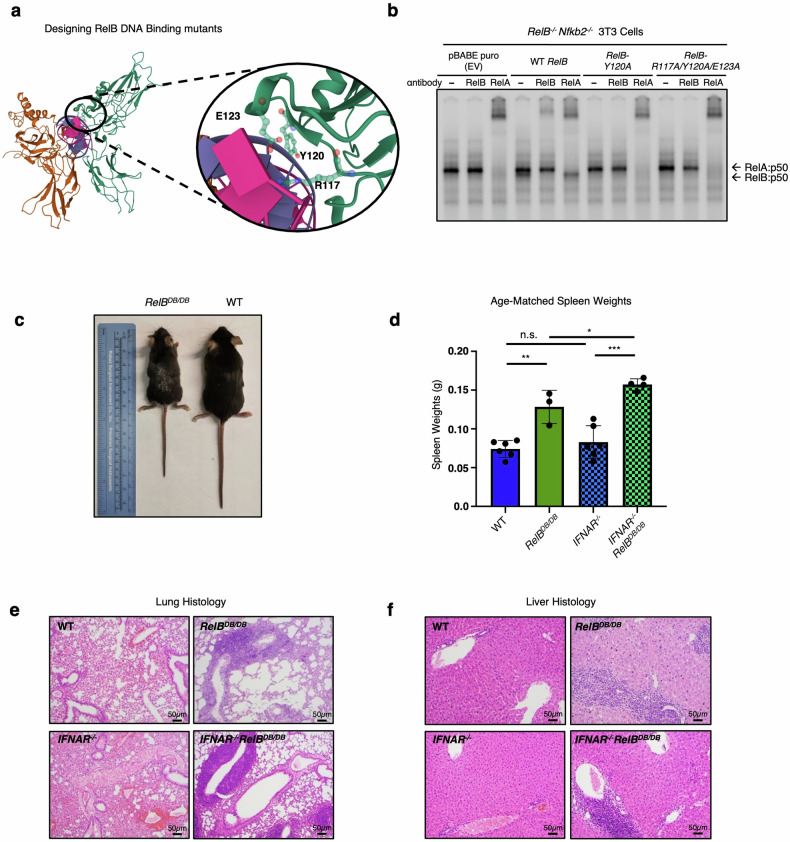
RelB requires its DNA binding function to suppress autoimmune pathology. **a** Crystal structure of the RelB DNA binding domain that guides the alanine substitutions at R117, Y120, and E123. **b** EMSA with pBABE puro (EV), *RelB*^*WT*^, *RelB*^*Y120A*^, and *RelB*^*DB/DB*^ reconstitution in *Relb*^−/−^*nfkb2*^−/−^ 3T3 cells stimulated 30 min with TNF (1 ng/mL). **c** Representative image of *RelB*^*DB/DB*^ mouse (left) and WT littermate (right) at 8 weeks of age; ruler for scale. **d** Spleen weights from age-matched WT (dark blue), *RelB*^*DB/DB*^ (dark green), *IFNAR*^−/−^ (light blue, checkered), *IFNAR*^−/−^*RelB*^*DB/DB*^ (light green, checkered) mice. ***P < 0.001, **P < 0.01, *P < 0.05, n.s., not significant; error bars indicate SD. Statistical analysis was done using an unpaired two-tailed Student’s *t*-test. **e**, **f** Representative images from H&E**-**stained sections of WT, *RelB*^*DB/DB*^, *IFNAR*^−/−^, and *IFNAR*^−/−^*RelB*^*DB/DB*^ lungs (**e**), and livers (**f**). *n* = 3–4.

These data suggest so far that RelB may suppress the NF-κB pro-inflammatory gene expression in BMDCs, and potentially the inflammatory pathology, via competition with RelA for binding to sites near the regulatory promoter regions of these genes. To test this hypothesis further, we generated a novel RelB mutant mouse strain (*RelB*^*DB/DB*^) with the targeted ablation of its high-affinity DNA binding. Using the previously reported crystal structure of RelB that reveals the protein–DNA binding interface^57^, we designed and characterized RelB variants with a single amino acid substitution (RelB^Y120A^) converting Tyr120 to alanine (Y120A), or a triple substitution within the amino-terminal DNA binding domain of RelB, converting Arg117, Tyr120, and Glu123 to alanine (R117A, Y120A, and E123A) (Fig. 4a). After we confirmed that the ectopic expression of these RelB protein variants was similar to that of WT RelB protein in RelB-deficient 3T3 cells (Supplementary Fig. S1), we measured their ability to bind DNA via electrophoretic mobility shift assay (EMSA). We found that WT RelB protein was able to bind κB sites, while both single RelB^Y120A^ and triple RelB^R117A/Y120A/E123A^ mutant proteins were unable to bind to κB sites, confirming the directed loss of DNA binding function (Fig. 4b). To ensure maximal reduction in DNA binding affinity, we decided to use the triple mutant design for generating the RelB DNA binding mutant mouse (*RelB*^*DB/DB*^).

Upon knockin embryonic stem cell generation, blastocyst complementation, germline transmission, and breeding to homozygosity, *RelB*^*DB/DB*^ mutant mice appeared runted and smaller than WT littermates. At 8 weeks of age they displayed a hunched posture, scaly skin, and enlarged abdomens, similar to *RelB*^−/−^ mice^17^ (Fig. 4c). *RelB*^DB/DB^ mice showed marked splenomegaly as reported in *RelB*^−/−^ mice, with spleens from *RelB*^*DB/DB*^ mice weighing on average 1.7× more than the spleens from WT controls (Fig. 4d). To determine whether this phenotype was type I IFN-dependent or -independent, we bred the new *RelB*^*DB/DB*^ mutant strain with the *IFNAR*^−/−^ mouse strain to produce a compound mutant. Similar to our previous findings^20^, splenomegaly was found to be independent of IFN signaling with spleens from *IFNAR*^−/−^*RelB*^*DB/DB*^ mice weighing more than spleens from WT and single *IFNAR*^−/−^ mice on average by 2.1- and 1.9-fold, respectively (Fig. 4d). Blinded histologic analysis of spleens from *RelB*^−/−^ and *IFNAR*^−/−^*RelB*^−/−^ mice revealed a severe contraction in white pulp, expansion of red pulp, and increased extramedullary hematopoiesis when compared with WT and *IFNAR*^−/−^ controls (Supplementary Figs. S2, S3), consistent with prior reports^17,20^. Analysis of spleens from *RelB*^*DB/DB*^ mice similarly displayed an intermediate contraction in the white pulp, expansion of red pulp, and increased extramedullary hematopoiesis compared to WT (Supplementary Figs. S2, S3). Spleens from *IFNAR*^−/−^*RelB*^*DB/DB*^ mice also revealed an intermediate contraction in white pulp; however, little to no expansion of the red pulp nor increase in extramedullary hematopoiesis was noted when compared with *IFNAR*^−/−^ controls (Supplementary Figs. S2, S3). In the lungs, blinded histologic analysis revealed moderate to marked perivascular mixed lymphocytic and neutrophilic infiltrates in *RelB*^−/−^ and *IFNAR*^−/−^*RelB*^−/−^ as previously reported^17,20^, while no significant inflammation was noted in WT and *IFNAR*^−/−^ controls (Fig. 4e; Supplementary Fig. S4). Lung histology in *RelB*^*DB/DB*^ and *IFNAR*^−/−^*RelB*^*DB/DB*^ mice similarly showed moderate to marked perivascular inflammation, yet contained predominantly lymphocytic infiltrates (Fig. 4e; Supplementary Fig. S4). Consistent with lung findings and prior studies^17,20^, blinded histologic analysis of livers from *RelB*^−/−^ and *IFNAR*^−/−^*RelB*^−/−^ mice revealed moderate to marked mixed lymphocytic/neutrophilic inflammation in periportal and centrilobular patterns (Fig. 4f; Supplementary Fig. S5). Again, *RelB*^*DB/DB*^ and *IFNAR*^−/−^*RelB*^*DB/DB*^ livers showed a similar degree of inflammation to *RelB*^−/−^ and *IFNAR*^−/−^*RelB*^−/−^ livers, but infiltrates were predominantly composed of lymphocytes (Fig. 4f; Supplementary Fig. S5). Thymi from *RelB*^*DB/DB*^ mice weighed less than WT controls, indicating thymic atrophy as seen in *RelB*^−/−^ mice (Supplementary Fig. S6). Thymi from *IFNAR*^−/−^*RelB*^*DB/DB*^ mice showed size variation intermediate between *RelB*^*DB/DB*^ and WT controls (Supplementary Fig. S6). Finally, in the serum, *RelB*^*DB/DB*^ and *IFNAR*^−/−^*RelB*^*DB/DB*^ mice showed elevated levels of both pro-inflammatory cytokines IP-10 and IL-6, consistent with elevated levels previously reported in *RelB*^−/−^ mice^20,22,58^ (Supplementary Fig. S7). Together, this phenotypic and histological analysis of the *RelB*^*DB/DB*^ mutant strain provides compelling evidence that the DNA-binding mutant of RelB fails to suppress critical aspects of the autoimmune pathology associated with the loss of RelB.

### RelB DNA binding mutant DCs hyper-activate pro-inflammatory gene expression

We then addressed whether the RelB DNA binding mutant is able to suppress the induction of IFN-dependent and/or IFN-independent pro-inflammatory genes that are hyper-expressed in *RelB*^−/−^ mice. We compared BMDCs derived from WT, *RelB*^−/−^, and *RelB*^*DB/DB*^, as well as *IFNAR*^−/−^, *IFNAR*^−/−^*RelB*^−/−^, and *IFNAR*^−/−^*RelB*^*DB/DB*^ compound mutant mice in which type I IFN signaling is ablated yet IFN-independent gene expression remains intact. We stimulated BMDCs from all genotypes, collected cells at several time points (0 h, 1 h, 3 h, and 8 h), and then performed RNA-seq and differential gene expression analysis.

**Fig. 5:**
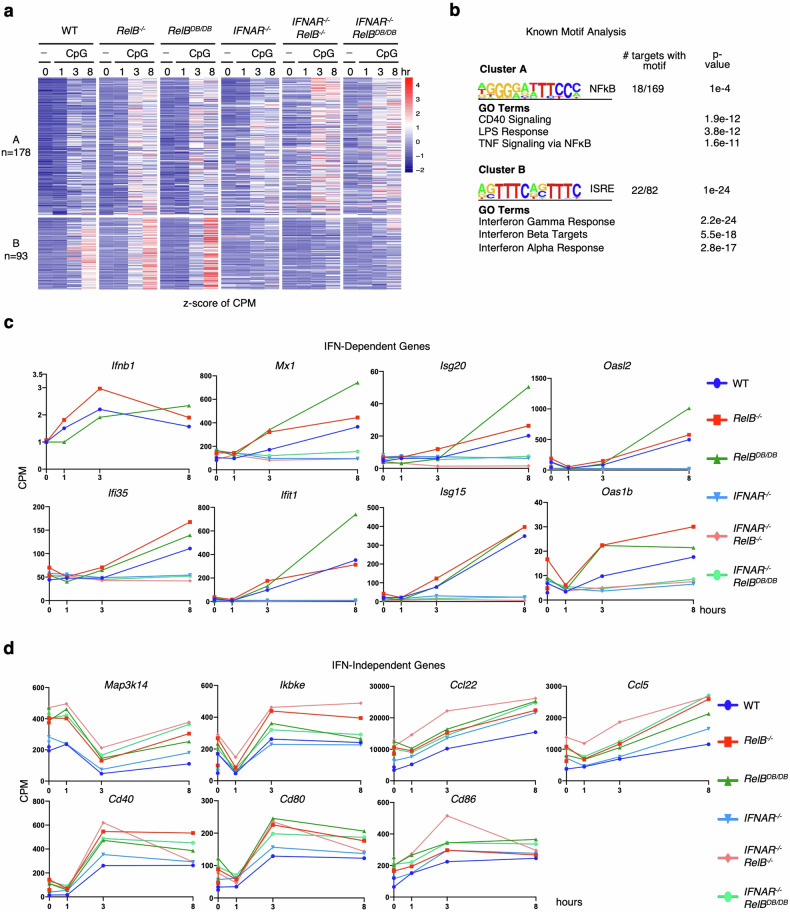
RelB suppresses type I IFN signaling and pro-inflammatory genes via its DNA binding function. **a** Heatmap of *z*-scored CPM of all *RelB*^−/−^ hyper-expressed genes in WT, *RelB*^−/−^, *RelB*^*DB/DB*^, *IFNAR*^−/−^, and *IFNAR*^−/−^*RelB*^−/−^, *IFNAR*^−/−^*RelB*^*DB/DB*^ BMDCs. Genes selected for CpG (0.1 µM) induced (log_2_FC > 1) in WT or *RelB* BMDCs at any time point and hyper-expressed (FC > 1.5) in *RelB*^−/−^ BMDCs relative to WT BMDCs at any time point (271 genes). Each row represents individual genes, and each column is from an individual time point upon CpG stimulation. **b** Top result of known motif analysis for gene clusters from **a**. Motif analysis considered –1 kb to +1 kb with respect to the TSS, GO results for gene clusters from **a**. **c** Line graphs of gene expression (CPM) for IFN-β and ISGs upon CpG stimulation (0 h, 1 h, 3 h, and 8 h). A dark blue line (circle) represents WT BMDCs, a dark red line (square) represents *RelB*^−/−^ BMDCs, dark green line (triangle) represents *RelB*^*DB/DB*^ BMDCs, light blue line (inverted triangle) represents *IFNAR*^−/−^ BMDCs, light red line (diamond) represents *IFNAR*^−/−^*RelB*^−/−^ BMDCs, and light green line (circle) represents *IFNAR*^−/−^*RelB*^*DB/DB*^ BMDCs. **d** Line graphs of gene expression (CPM) for IFN-independent hyper-expressed genes during CpG stimulation (0 h, 1 h, 3 h, and 8 h). A dark blue line (circle) represents WT BMDCs, a dark red line (square) represents *RelB*^−/−^ BMDCs, a dark green line (triangle) represents *RelB*^*DB/DB*^ BMDCs, the light blue line (inverted triangle) represents *IFNAR*^−/−^ BMDCs, light red line (diamond) represents *IFNAR*^−/−^*RelB*^−/−^ BMDCs, and light green line (circle) represents *IFNAR*^−/−^*RelB*^*DB/DB*^ BMDCs.

To identify genes that were hyper-expressed by the complete loss of RelB, we selected transcripts that were both induced by CpG (0.1 µM) (log_2_FC > 1) in either WT or *RelB*^−/−^ BMDCs and hyper-expressed (FC > 1.5) in *RelB*^−/−^ BMDCs relative to WT BMDCs. We then plotted the expression of these genes in BMDCs derived from WT, *RelB*^−/−^, *RelB*^*DB/DB*^, *IFNAR*^−/−^, *IFNAR*^−/−^*RelB*^−/−^, and *IFNAR*^−/−^*RelB*^*DB/DB*^ mice (Fig. 5a). Using k-means clustering, we identified an IFN-dependent hyper-expressed cluster (B; 93 genes) containing genes hyper-expressed in *RelB*^−/−^ BMDCs (average fold differences of 1.25× to 1.5× compared to WT BMDCs at all observed time points), with absent induction in all *IFNAR*^−/−^ containing BMDCs (average fold differences of 0.5× to 0.6× in all *IFNAR*^−/−^ containing BMDCs compared to WT BMDCs at the late 8 h stimulation time point) (Fig. 5a; Supplementary Table S4). Notably, cluster B genes were also hyper-expressed in BMDCs derived from *RelB*^*DB/DB*^ mice, with fold differences ranging from 1.35× to 1.5× between *RelB*^*DB/DB*^ and WT BMDCs at all observed time points (Fig. 5a). As expected, motif analysis of cluster B produced the IFN sensitive response element (ISRE) as the top statistically enriched motif, and GO analysis revealed IFN signaling pathways “interferon beta” and “interferon alpha” among the top terms (Fig. 5b). Individual genes in this IFN-dependent hyper-expressed cluster B included established ISGs such as *Mx1*, *Isg20*, *Oasl2*, *Ifi35*, *Ifit1*, *Isg15*, *Oaslb*, all of which were all also hyper-expressed in BMDCs derived from *RelB*^*DB/DB*^ mice but not in BMDCs derived from compound *IFNAR*^−/−^*RelB*^*DB/DB*^ mice (Fig. 5c). Notably, IFN-dependent genes previously described to be hyper-expressed in *RelB*^−/−^ BMDCs in response to polyI:C did not show a consistent hyper-expression phenotype in BMDCs derived from *RelB*^*DB/DB*^ mice^20^ (Supplementary Fig. S8), indicating slight differences in the patterns of IFN-dependent gene expression in *RelB*^−/−^ and *RelB*^*DB/DB*^ DCs upon CpG vs polyI:C stimulation.

Analysis of cluster A (178 genes) identified genes that were hyper-expressed in all *RelB*^−/−^ containing genotypes, but were not hyper-expressed in BMDCs derived from single *IFNAR*^−/−^ mutant mice, consistent with previously established IFN-independent pro-inflammatory genes^20^. BMDCs derived from the *RelB*^*DB/DB*^ and *IFNAR*^−/−^*RelB*^*DB/DB*^ compound mutant mice showed similar induction and hyper-expression of cluster A genes to BMDCs derived from *RelB*^−/−^ and *IFNAR*^−/−^*RelB*^−/−^ mice (Fig. 5a). Specifically, cluster A genes showed average fold differences ranging from 1.3× to 1.5× between *RelB*^*DB/DB*^ BMDCs and WT BMDCs, 1.6× to 2.0× between *IFNAR*^−/−^*RelB*^*DB/DB*^ BMDCs and WT BMDCs, and 1.2× to 1.3× between *IFNAR*^−/−^*RelB*^*DB/DB*^ BMDCs and single *IFNAR*^−/−^ BMDCs at all observed time points (Fig. 5a; Supplementary Table S4). Motif analysis of cluster A genes detected NF-κB as the top statistically enriched motif, and GO analysis revealed NF-κB activating pathways “CD40”, “LPS” and “TNF-alpha signaling” among the top GO terms (Fig. 5b). Notably, genes within this IFN-independent cluster A included *Map3k14*, *Ikbke*, *Ccl22*, *Ccl5*, *Cd40*, *Cd80*, and *Cd86*, which are NF-κB-inducible pro-inflammatory genes and previously shown to be hyper-expressed in *RelB*^−/−^ and *IFNAR*^−/−^*RelB*^−/−^ BMDCs^20^. Here, we found that these genes were also hyper-expressed in *RelB*^*DB/DB*^ and *IFNAR*^−/−^*RelB*^*DB/DB*^ BMDCs relative to WT and single *IFNAR*^−/−^ BMDCs (Fig. 5c, d). Stimulation with polyI:C showed similar effects (Supplementary Fig. S9). Together, these data provide strong evidence that RelB suppresses IFN-independent pro-inflammatory gene expression via direct binding to DNA and competition with RelA for κB sites.

## Discussion

Here we aimed to elucidate the mechanism by which RelB suppresses the hyper-expression of pro-inflammatory genes and inflammatory histopathology seen in *RelB*^−/−^ mice^17,20^. We found that the RelB-deficient pathology is IFN-independent, as assessed in the lung, liver, and thymus. As previous studies have pinpointed the loss of RelB in DCs alone to be sufficient in driving inflammatory pathology in the liver, spleen, lung, and thymi of *RelB*^−/−^ mice^21–23^, we investigated dysregulated gene regulatory mechanisms in *RelB*^−/−^ DCs. Our RNA-seq and ChIP-seq analyses of BMDCs from *RelB*^−/−^ mice revealed elevated binding by RelA to a subset of genomic κB sites that were bound by RelB in resting WT BMDCs. Our analyses revealed that elevated binding by RelA to the promoter regions of known IFN-independent pro-inflammatory genes was correlated with their hyper-expression. These data support the hypothesis that RelB inhibits the expression of pro-inflammatory genes by competing with RelA for binding to their promoter regions.

To further test this hypothesis, we generated a novel *RelB*^*DB/DB*^ mouse containing mutations of three specific amino acids that abrogate κB site-specific DNA binding. Indeed, we found that the functional loss of RelB DNA binding resulted in the hyper-expression of IFN-independent pro-inflammatory genes in BMDCs similar to the hyper-inflammatory phenotype seen by the complete loss of RelB^20^. Further, *RelB*^*DB/DB*^ mutant mice appeared runted and smaller than WT littermates at 8 weeks of age, similar to the *RelB*^−/−^ mice^17^. *RelB*^*DB/DB*^ mice displayed hunched posture, scaly skin, enlarged abdomens, thymic atrophy, and splenomegaly like *RelB*^−/−^ mice^17,20^. Further, *RelB*^*DB/DB*^ mice revealed moderate to marked perivascular inflammation in the lungs, and moderate to marked periportal and centrilobular inflammatory infiltrates in the liver. Like *RelB*^−/−^ mice, these hallmarks of the inflammatory pathology were not rescued by the generation of *IFNAR*^−/−^*RelB*^*DB/DB*^ compound mutant mice. Notably, while *RelB*^−/−^ and *IFNAR*^−/−^*RelB*^−/−^ mice showed mixed lymphocytic and neutrophilic infiltrates in both the lung and liver as previously reported, *RelB*^*DB/DB*^ and *IFNAR*^−/−^*RelB*^*DB/DB*^ mice instead displayed predominantly lymphocytic infiltrates in both the lung and liver, suggesting that while *RelB*^−/−^ and *RelB*^*DB/DB*^ mice share a common primary auto-inflammatory mechanism, there are likely compounding mechanisms caused by the complete loss of RelB leading to additional neutrophilic infiltrates and slight variations in spleen pathology.

The literature provides a rich set of potential molecular mechanisms by which RelB controls gene expression, ranging from altering IKK activity, to stabilization of the repressive p100/p105 complex, to DNA methylation and establishing repressive histone modifications, to simple competition with RelA for κB sites^37^^,38,41,45–47^^,59,60^. Our RelA/RelB ChIP-seq and RNA-seq studies suggest that competition between the two NF-κB family members is the key mechanism, and the fact that the RelB DNA binding mutant near-phenocopies the RelB knockout provides further support for this simple model of balanced NF-κB target gene expression control.

Our findings do not determine the exact function of RelB upon binding to DNA and do not rule out the involvement of cytoplasmic functions. Epigenomic profiling of the repressive H3k9me2 modification did not reveal RelB-dependence even though SMAC mimetics applied to DCs, which induce RelB among other signaling pathways, led to statistically significant changes (data not shown). Further biochemical characterization of DCs derived from the RelB DNA binding mutant may also reveal additional gene regulatory mechanisms. It is worth noting that the *Nfkb2* p100 protein, which has the potential to limit how much RelA-containing dimer may be activated, has also been reported as an inhibitor of DC activation, with *Nfkb2*^−/−^ DCs showing elevated MHC class II and costimulatory molecule expression and an enhanced ability to induce CD4^+^ T cell responses^61^. However, the autoimmune pathology is sporadic in only some mice and is mild, with the great majority having unaffected lifespans^62–65^. These data support our conclusion that the stabilization of p100 function by RelB is not the key mechanism leading to lethal multi-organ pathology in *RelB*^−/−^ mice.

Further, while our mechanistic studies were performed in DCs, our phenotypic characterization of the autoimmune pathology was done utilizing mice with a global RelB DNA binding mutant. While this mutant may also have defective functions in other immune cells such as medullary thymic epithelial cells (mTECs), which have also been implicated in the RelB knockout pathology, previous work has demonstrated that correcting RelB deficiency in DCs is sufficient to restore appropriate mTEC and T-regulatory (Treg) cell numbers in the thymus^23^ and improve autoinflammatory pathology^21^, suggesting that DC pathology is causal of changes in the thymic niche. However, whether this niche-modulatory function of RelB in DCs is dependent on high-affinity DNA binding remains unknown. Therefore, further studies of how DCs derived from the RelB DNA binding mutant influence other immunomodulatory cell types in autoimmune pathology may be warranted.

The concept of NF-κB dimers competing for κB sites also applies to the p50 subunit, which on the one hand, is a dimerization partner for RelA, but on the other hand, may homodimerize and have antagonistic effects on RelA-mediated gene activation. Given that p50 homodimers lack a transactivation domain^66^, but are able to bind DNA and compete with RelA:p50 for binding to κB sites at regulatory regions, p50 homodimers are thought to act as repressors of NF-κB gene expression. Indeed, overexpression of p50 was shown to suppress RelA-induced expression of several immune response genes such as *H2-K1*, *ICAM1*, and *IL2*^67–69^. The p50 homodimer was also reported to interfere with the binding of IFN regulatory factors on ISREs that contain a triple G motif (e.g., GGGRA), thus resembling a half-site of the palindromic κB element^70^.

How could RelB function both as a transcriptional activator for organogenic chemokine and metabolic genes^33^^,54^^,71^^,72^ and as a transcriptional repressor for pro-inflammatory genes^32^? One possibility is that RelB’s role is determined by its dimerization partner, whether it is p50 or p52. As a RelB:p50 dimer, RelB may have similar DNA binding specificity to RelA:p50^55^ and thus compete with the RelA:p50, which is a strong pro-inflammatory transcriptional activator. However, p52 has been shown to confer a different DNA sequence specificity to NF-κB dimers^15,73^. Thus, the RelB:p52 heterodimer, a weaker transcriptional activator, may be binding to promoter elements that are not accessed by RelA:p50, such as on organogenic chemokine genes^74^. Differences in mRNA half-life in the two sets of genes may amplify the specificity due to RelA:p50’s transient and RelB:p52’s long-lasting activity dynamics. Further studies are needed to substantiate this conceptual framework.

Our present findings highlight that NF-κB RelA transcriptional activity, once released from IκB proteins, is further finetuned by RelB in accessing the κB sites of target genes. As such, we provide mechanistic insights into the autoinflammatory mechanisms that drive RelB-deficient pathology. While substantial inflammatory consequences of RelB deficiency are seen phenotypically, our findings suggest that they are caused by small changes in RelA binding to target gene promoters and target gene expression in DCs. As such, RelB-deficient pathology and other relopathies may likely require therapeutic approaches focused on finetuning these slight changes in dysregulated gene regulatory mechanisms. Given the extensive list of pathologies linking NF-κB to autoimmunity and dysregulation of IFN-dependent and IFN-independent pro-inflammatory gene expression^6,7,8^, the present analysis may inform ongoing efforts to elucidate the molecular mechanisms that mediate them.

## Materials and Methods

### Design of RelB DNA binding mutants

The pBABE-puro vector containing RelB variants was constructed by first amplifying the coding region of murine *RelB* cDNA corresponding to amino acids 1–558 by PCR, and inserting it into the *Eco*RI and *Bam*HI sites of the polylinker. Mutagenesis using Agilent QuikChange II Site-Directed Mutagenesis Kit (Agilent Technologies) was performed for RelB point mutations. DNA binding mutants within the amino-terminal of RelB were generated by making single or triple substitutions at residues Arg117, Tyr120, and Glu123 to Ala (R117A, Y120A, and E123A). RelB-Y120A was obtained by PCR using the oligonucleotides (5′-tggcatgcgcttccgcGCcgagtgcgagggccgc-3′ and 5′-gcggccctcgcactcgGCgcggaagcgcatgcca-3′). RelB-R117A/Y120A/E123A mutant was obtained using the oligonucleotides (5′-cagcgtggcatgGCcttccgcGCcgagtgcGCgggccgctcggcc-3′ and 5′-ggccgagcggcccGCgcactcgGCgcggaagGCcatgccacgctg-3′).

### Generation of cell lines harboring RelB DNA binding mutants

Immortalized *Relb*^−/−^*nfkb2*^−/−^ fibroblast cells were cultured in Dulbecco’s Modified Eagle’s Medium (DMEM) supplemented with 10% bovine calf serum (BCS), 1% penicillin-streptomycin and 1% l-glutamine. Platinum-E (Plat-E) cells were cultured in 10% fetal calf serum, 1% penicillin-streptomycin, 1% l-glutamine, and supplemented with 1 μg/mL puromycin and 10 μg/mL of blasticidin. For transfection, Plat-E retroviral packaging cell line^75^, was plated on 10-cm plates 16 h prior at 50% confluency in DMEM supplemented with 10% fetal calf serum, 1% penicillin-streptomycin, and 1% l-glutamine. Cells were transfected using 300 μL of Opti-MEM medium (Thermo Fisher Scientific) and polyethylenimine (PEI; 1 μg/μL in 1× PBS, pH 4.5, Polysciences #23966-2) with 7 μg of retroviral construct DNA, pBABE-puro empty vector (EV control) or RelB-expressing constructs (4:1 ratio of PEI (μL):plasmid DNA (μg)). The transfection complex (Opti-MEM medium, PEI reagent, plasmid DNA) was incubated for 15 min at room temperature, then added drop-wise to Plat-E cells, and incubated for 6 h. Transfection media was then replaced with fresh DMEM containing 10% fetal calf serum, 1% penicillin-streptomycin, and 1% l-glutamine. Cells were further incubated for a total of 48 h prior to collecting viral supernatants. Virus-containing supernatant was filtered through a 0.45-μm filter and used to infect *RelB*^−/−^*nfkb2*^−/−^ fibroblast cells with the addition of 4 μg/mL Polybrene. 48 h post-infection of cells with viruses, the stably transduced cells were then selected with 2.5 μg/mL puromycin for a total of 72 h. After selection with puromycin, the puromycin-containing medium was removed and cells were passaged twice for recovery prior to being expanded in culture for experiments. EV, pBABE-puro vector without the *RelB* gene fragment, is used as a negative control, to maintain a stable retrovirally transduced RelB knockout cell line, meanwhile, pBABE-puro containing full-length RelB (RelB-WT) is used as a positive control for RelB-expressing cell line.

### Biochemical characterization of RelB DNA binding mutants

Western blotting and EMSA were performed with standard methods as described previously^54,76,77^. Western blots were probed with antibodies specific for RelB (Santa Cruz Biotechnology, Cat# sc-226), and Actin (Santa Cruz Biotechnology, Cat# sc-1615). For EMSA, fibroblast cells were stimulated with 1 ng/mL TNF, nuclear extracts were harvested at the indicated times (h), and previously published protocols were followed^78,79^.

### Generation of the RelB DNA binding mutant mouse strain

The *RelB*^*DB/DB*^ mutant mouse line was generated by Ingenious Targeting Laboratory (iTL, www.genetargeting.com) using the targeted Neomycin selection cassette for the conventional knockin model. A donor sequence encoding the mutant RelB protein was used to generate, via homologous recombination, a tagged embryonic stem cell line carrying a Neo resistance marker, that was implanted to yield heterozygous mice. These mice were then bred with a mouse line constitutively expressing the Flp recombinase to remove the Neo resistance marker included in the homologous donor sequence. We then back-crossed the resultant mice with WT C57BL/6J mice to remove the Flp background and generate the completed, germline heterozygous mutant knockin line. Further, a subsequent round of heterozygous mating was performed to obtain the homozygous knockin of *RelB*^*DB/DB*^ mice.

### Mouse husbandry

WT, knockout, and knockin mice were housed in pathogen-free conditions at the University of California, Los Angeles. *RelB*^−/−^ mice were generated by breeding *RelB*^*+/*–^ mice, *IFNAR*^−/−^*RelB*^−/−^ mice were generated by breeding *IFNAR*^−/−^*RelB*^*+/*–^ or *IFNAR*^*+/–*^*RelB*^*+/*–^ mice, and *RelB*^*DB/DB*^ mice were generated by breeding *RelB*^*+/DB*^ mice. All mice used for experiments were between 4 and 12 weeks on the day of the experiment, both male and female mice were used for experiments.

### BMDCs

Bone marrow cells were isolated from mouse femurs and cultured with 20 ng/mL GM-CSF and 10 ng/mL IL-4 to produce BMDCs with half the media being replaced on days 3 and 6 as previously reported^38^. Cells were stimulated with CpG (0.1 µM) (Invivogen ODN 1668, Cat# tlrl-1668) or Poly(I:C) HMW (10 μg/mL) (Invivogen; Cat# tlrl-pic) and collected at specified time points in Invitrogen™ TRIzol™ Reagent (Cat# 15-596-018) for RNA and was extracted using Qiagen RNeasy Mini Kit (Cat# 74106) as described^54^.

### Transcriptome profiling

RNA was used for RNA-seq as described^80^. Briefly, libraries were prepped using the KAPA Stranded mRNA-Seq Kit Illumina® platform (KR0960-v3.15) using 1 µg of RNA per sample measured using a Qubit 2.0 fluorometer. Final libraries were checked via agarose gel and multiplexed with a maximum of 24 samples per sequencing run. Libraries were sequenced using Illumina HiSeq 3000 with single-end 50-bp reads at the UCLA Technology Center for Genomics & Bioinformatics.

### Bioinformatic analysis

Reads were trimmed using cutadapt (1) (cutoff *q* = 20) and mapped to the mm10 genome. Processed reads showed high-quality reads and alignment scores. The October 2014 version of the Ensembl database was used to extract gene annotation information. CPM values were generated using edgeR (5) to normalize the raw count data based on sequencing depth. To permit FC calculations, a pseudocount of 1 CPM was added. Induced genes were selected using a log_2_FC > 1 cutoff for any stimulated time point relative to 0 h unstimulated control, and transcripts with empty gene names were removed. Data were *z*-scored and plotted using the pheatmap R package. Fold differences of genes within heatmaps were calculated by first calculating the fold differences for all individual genes between genotypes of interest and WT or *IFNAR*^−/−^ control CPM (Genotype X)/CPM (Genotype Y) for each individual time point. Average fold differences were then calculated by averaging the fold differences of all genes within each cluster for each individual time point. GO and motif analysis was done via Homer suite considering regulatory regions within −1 kb to 1 kb from the TSS. Line graphs of individual genes were generated using GraphPad Prism.

### ChIP sample generation and data preprocessing

ChIP-seq was performed as previously described^81,82^ with anti-RelA antibody (Cell Signaling Technology, D14E12 or Cat# 8242) or RelB antibody (Cell Signaling Technology, D7D7W or Cat# 10544). Approximately 1 × 10^7^ BMDCs were used per sample. Two chemical crosslinkers, DSG and PFA, were used during sample preparation at concentrations of 1.0 mM and 1.0%, respectively. Sonication was performed on a Covaris M220-focused ultrasonicator. ChIP-seq libraries were prepared using KAPA HyperPrep Kits (Roche) and sequenced using Illumina HiSeq 3000 with single-end 50-bp reads at the UCLA Technology Center for Genomics & Bioinformatics. Reads were aligned using Hisat2 to the mouse genome (NCBI37/mm9). Peaks were called if they were enriched compared to input samples and had a false discovery rate of < 0.01 using HOMER software^83^. To compare peaks across samples, a master probe which contained all peaks from every sample was generated using BEDTools^84^. Then SeqMonk was used to find raw read counts for each peak in the master probe. RPKMs were generated using the length of each peak and the depth of sequencing for each sample.

### Tissue isolation, fixation, and scoring

Spleens, lungs, and livers were isolated from age-matched mice immediately after being euthanized and subsequently rinsed with PBS. After the removal of excess PBS, spleens were weighed. Fixation was done in 10% formaldehyde for 46–48 h. Tissue was processed, sectioned, and H&E-stained by the UCLA Translational Pathology Core Laboratory (TPCL). Histology slides were blinded and scored. Red pulp expansion was quantified and scored as adequate = 0, expanded = 1, and markedly expanded = 2. White pulp contraction was quantified and scored as adequate = 0, reduced = 1, and markedly reduced = 2. Extramedullary hematopoiesis was scored as present = 1, increased = 2, marked = 3. Lung and liver inflammation was quantified and scored as absent = 0, scant = 1, mild = 2, moderate = 3, and marked = 4. Lymphocyte and neutrophilic infiltrates were scored as absent = 0, scant = 1, mild = 2, moderate = 3, marked = 4.

## Supplementary information

Supplementary Figures

Supplementary Table S1

Supplementary Table S2

Supplementary Table S3

Supplementary Table S4

## Data Availability

The experimental data is available on the Gene Expression Omnibus (GSE236533) and is available in Supplementary information.
