# Supplementary material for: NF-κB RelB suppresses the inflammatory gene expression programs of dendritic cells by competing with RelA for binding to target gene promoters
Source: Cell Discov. 2025 Feb 11;11:13. doi: 10.1038/s41421-024-00767-9 (PMC11811218; doi:10.1038/s41421-024-00767-9)
Supplement: Supplementary file 1 — Supplementary Figures [file 41421_2024_767_MOESM1_ESM.pdf]

Supplemental Figures

Figure S1

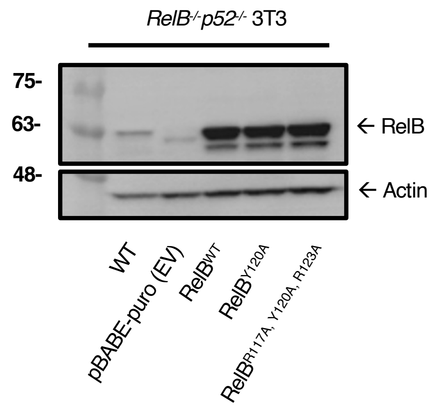

Figure S1: RelB variants have similar protein stability as indicated by expression level. Western Blot for RelB and Actin of unstimulated whole cell lysates from *Relb*$^{-/-}$ 3T3 cells reconstituted with pBABE-puro empty vector (EV), *RelB*^{WT}, *RelB*^{Y120A}*,* and *RelB*^{R117A,Y120A,E123A}.

Figure S2

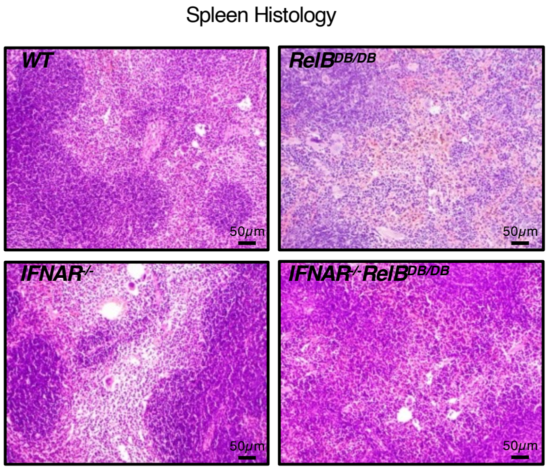

Figure S2: *RelB*$^{DB/DB}$ and *IFNAR*$^{-/-}$*RelB*$^{DB/DB}$ mice show similar spleen pathology. Representative images from H&E stained sections of *WT*, *RelB*$^{DB/DB}$, *IFNAR*$^{-/-}$, and *IFNAR*$^{-/-}$ *RelB*$^{DB/DB}$ spleen. Scale bar indicates 50µm; n=3-4

Figure S3

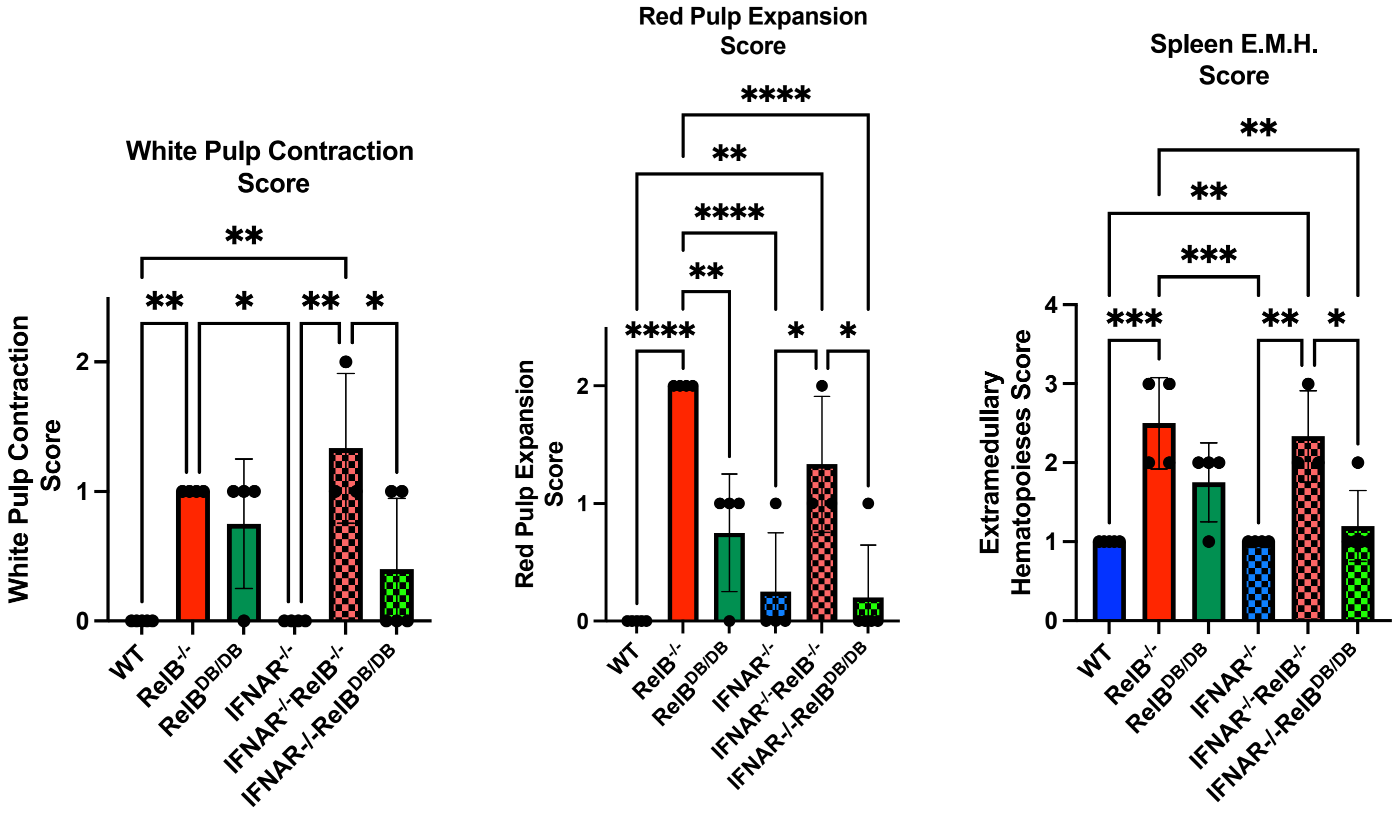

Phenotype Scores

Red pulp expansion		White pulp contraction		EMH	
adequate	0	adequate	0	present	1
expanded	1	reduced	1	increased	2
markedly expanded	2	markedly reduced	2	markedly increased	3

Figure S3: Spleen pathology of WT, *RelB*$^{-/-}$, *RelB*$^{DB/DB}$, *IFNAR*$^{-/-}$, *IFNAR*$^{-/-}$ *RelB*$^{-/-}$, and *IFNAR*$^{-/-}$ *RelB*$^{DB/DB}$ mice.

Bar graphs of quantification of blinded analysis of spleen pathology. Red pulp expansion scored as adequate= 0, expanded= 1, markedly expanded =2. White pulp contraction quantified as adequate= 0, reduced= 1, markedly reduced =2. Extramedullary hematopoiesis scored as present= 1, increased=2, marked=3. WT (dark blue), *RelB*$^{-/-}$ (dark red), *RelB*$^{DB/DB}$ (dark green), *IFNAR*$^{-/-}$(light blue, checkered), *IFNAR*$^{-/-}$*RelB*$^{-/-}$ (light red, checkered), *IFNAR*$^{-/-}$*RelB*$^{DB/DB}$ (light green, checkered) mice. (****=p<.0001,***=p<.001,**=p<.01 ,*=p<.05, absent= n.s.); error bars indicate S.D. Statistical analysis was done using one way ANOVA.

Figure S4

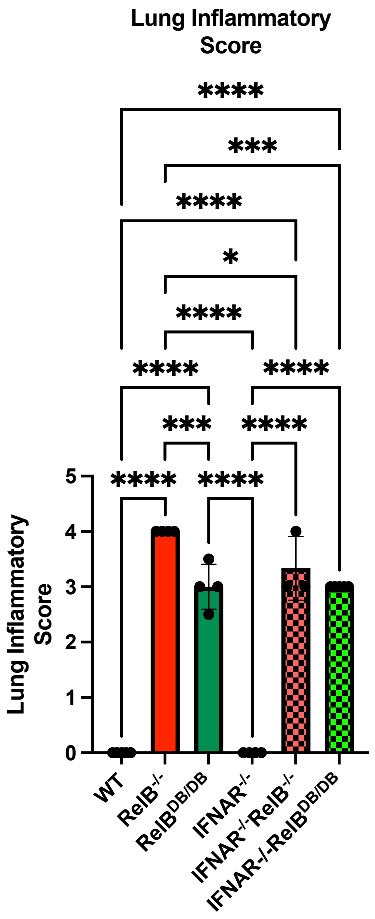

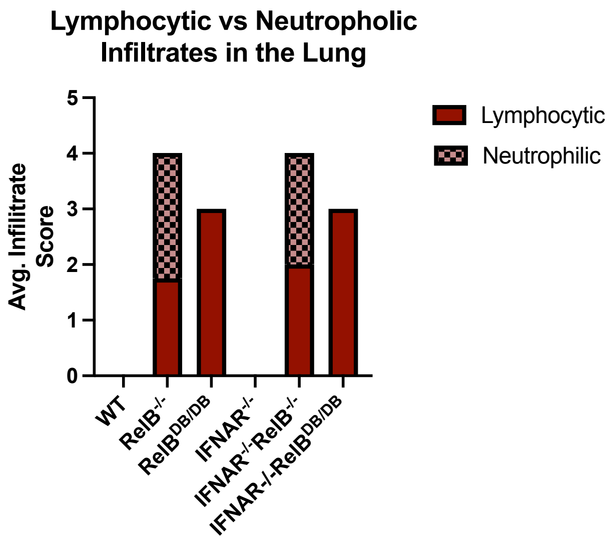

Infiltrate Scores

absent	0
scant	1
mild	2
moderate	3
marked	4

Figure S4: Lung inflammatory pathology of WT, *RelB$^{-/-}$*, *RelB$^{DB/DB}$*, *IFNAR$^{-/-}$*, *IFNAR$^{-/-}$RelB$^{-/-}$*, and *IFNAR$^{-/-}$RelB$^{DB/DB}$* mice.

Bar graphs of quantification of blinded analysis of lung pathology. Lung inflammation scored as absent= 0, scant= 1, mild =2, moderate=3, marked =4. Lymphocyte and neutrophilic infiltrates scored as absent= 0, scant= 1, mild =2, moderate=3, marked =4. WT (dark blue), *RelB$^{-/-}$* (dark red), *RelB$^{DB/DB}$* (dark green), *IFNAR$^{-/-}$*(light blue, checkered), *IFNAR$^{-/-}$RelB$^{-/-}$* (light red, checkered), *IFNAR$^{-/-}$RelB$^{DB/DB}$* (light green, checkered) mice. (****=p<.0001,***=p<.001,**=p<.01, *=p<.05, absent= n.s.); error bars indicate S.D. Statistical analysis was done using one way ANOVA.

Figure S5

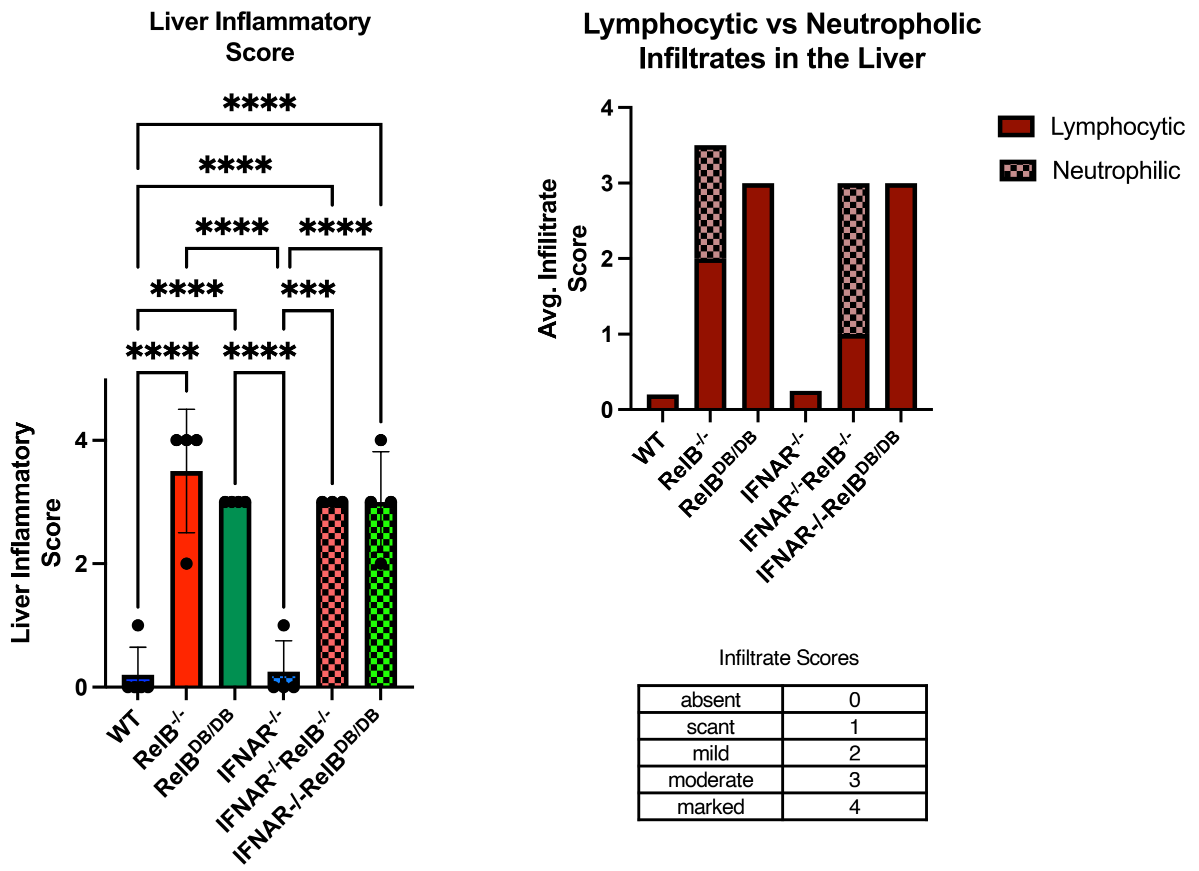

absent	0
scant	1
mild	2
moderate	3
marked	4

Figure S5: Liver inflammatory pathology of WT, *RelB$^{-/-}$*, *RelB$^{DB/DB}$*, *IFNAR$^{-/-}$*, *IFNAR$^{-/-}$RelB$^{-/-}$*, and *IFNAR$^{-/-}$RelB$^{DB/DB}$* mice.

Bar graphs of quantification of blinded analysis of liver pathology. Liver inflammation scored as absent= 0, scant= 1, mild =2, moderate=3, marked =4. Lymphocyte and neutrophilic infiltrates scored as absent= 0, scant= 1, mild =2, moderate=3, marked =4. WT (dark blue), *RelB$^{-/-}$* (dark red), *RelB$^{DB/DB}$* (dark green), *IFNAR$^{-/-}$* (light blue, checkered), *IFNAR$^{-/-}$RelB$^{-/-}$* (light red, checkered), *IFNAR$^{-/-}$RelB$^{DB/DB}$* (light green, checkered) mice. (****=p<.0001,***=p<.001,**=p<.01, *=p<.05, absent= n.s.); error bars indicate S.D. Statistical analysis was done using one way ANOVA.

Figure S6

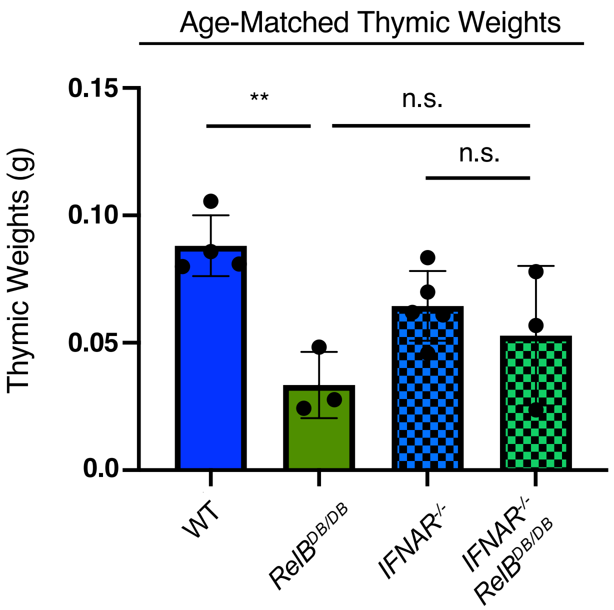

Figure S6: *RelB$^{DB/DB}$* mice show reduced thymi similar to *RelB$^{-/-}$* mice.
Bar graphs of thymic weights from age-matched *WT* (dark blue), *RelB$^{DB/DB}$* (dark green), *IFNAR$^{-/-}$*(light blue, checkered), *IFNAR$^{-/-}$RelB$^{DB/DB}$* (light green, checkered) mice. (**=p<.01, n.s.= not significant); error bars indicate S.D. Statistical analysis was done using unpaired 2-tailed students t-test.

Figure S7

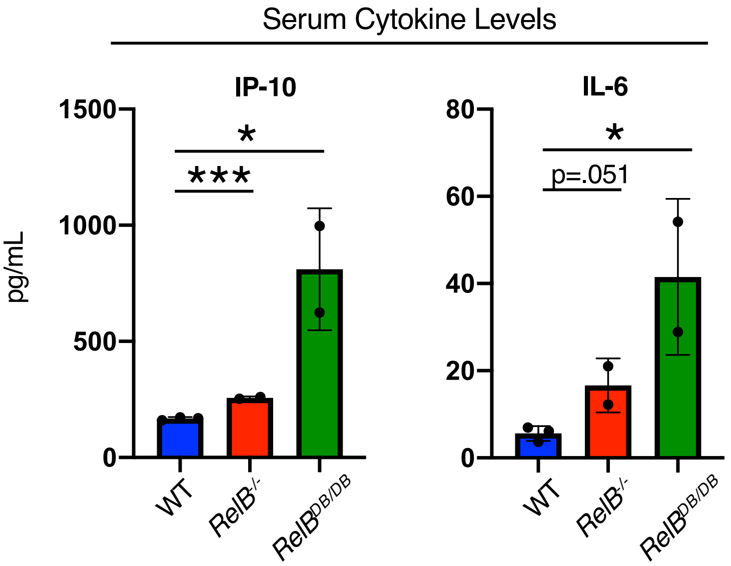

Figure S7: Serum levels of IL-6 and IP-10 cytokines in the blood of WT, *RelB$^{-/-}$*, *RelB$^{DB/DB}$* mice.
Bar graphs of quantification of serum levels of IL-6 and IP-10 protein. WT (dark blue), *RelB$^{-/-}$* (dark red), *RelB$^{DB/DB}$* (dark green) mice. (***=p<.001, *=p<.05); error bars indicate S.D. Statistical analysis was done using unpaired 2-tailed students t-test.

Figure S8

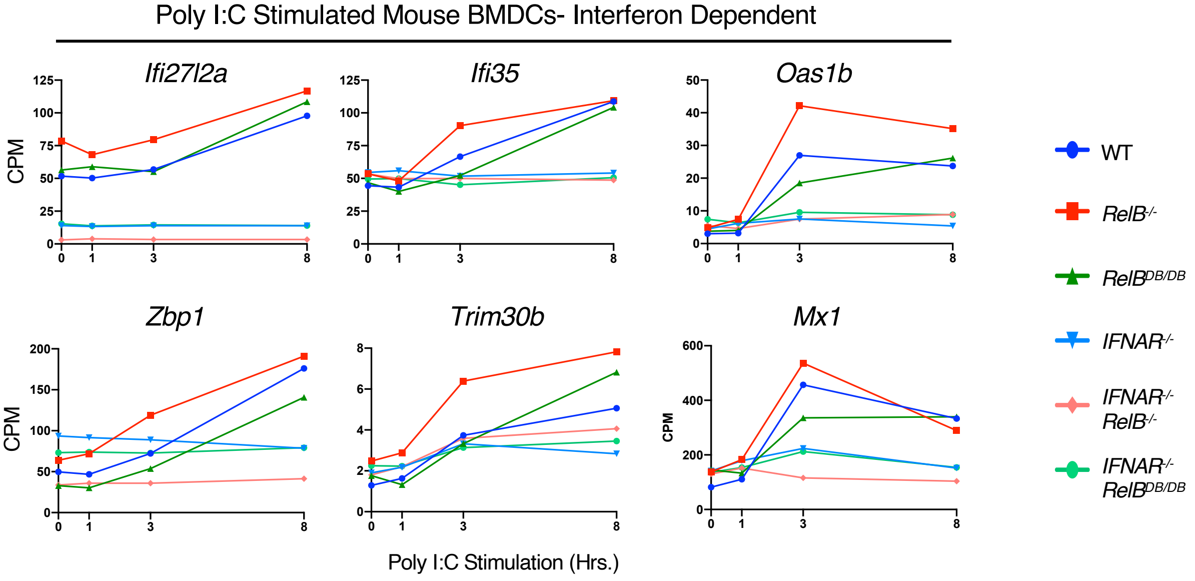

Figure S8: IFN-dependent gene expression in poly I:C stimulated BMDCs derived from WT, *RelB$^{-/-}$*, *RelB$^{DB/DB}$*, *IFNAR$^{-/-}$*, *IFNAR$^{-/-}$ RelB$^{-/-}$*, and *IFNAR$^{-/-}$ RelB$^{DB/DB}$* mice.

Line graphs of gene expression (CPM) for interferon stimulated genes upon poly I:C-stimulation (0,1,3, and 8hr) dark blue line (circle) represents *WT* BMDCs, dark red line (square) represents *RelB$^{-/-}$* BMDCs, dark green line (triangle) represents *RelB*$^{DB/DB}$ BMDCs, light blue line (inverted triangle) represents *IFNAR$^{-/-}$* BMDCs, light red line (diamond) represents *IFNAR$^{-/-}$RelB$^{-/-}$* BMDCs, light green line (circle) represents *IFNAR$^{-/-}$RelB*$^{DB/DB}$ BMDCs.

Figure S9

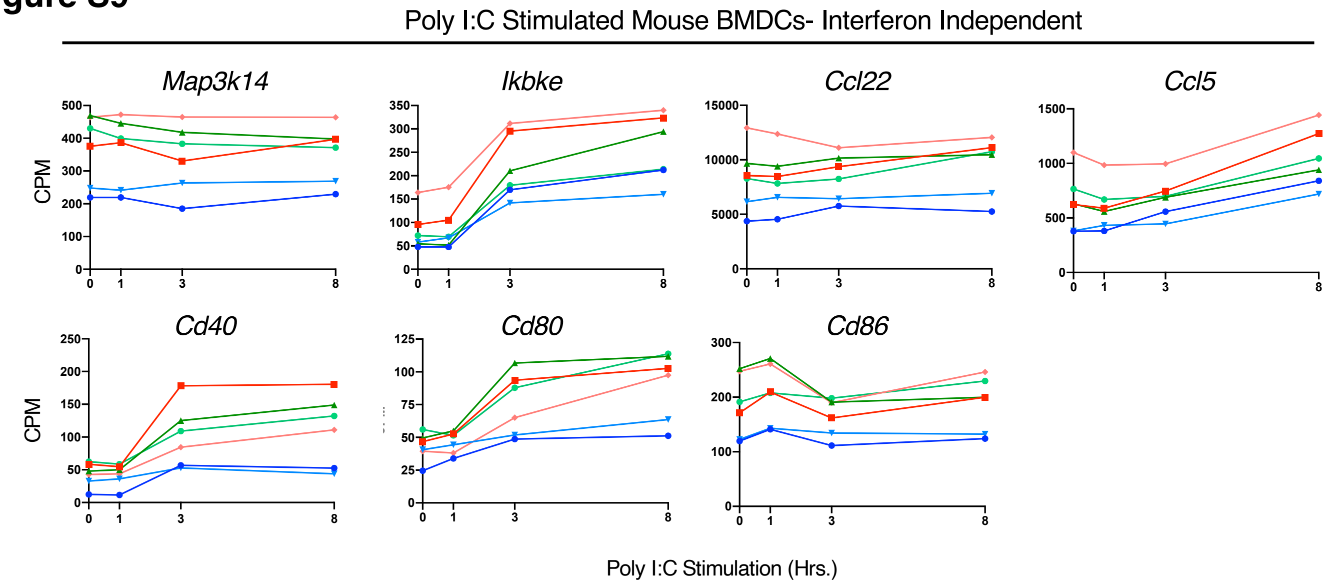

Figure S9: IFN-independent pro-inflammatory gene expression in poly I:C stimulated BMDCs derived from WT, *RelB$^{-/-}$*, *RelB$^{DB/DB}$*, *IFNAR$^{-/-}$*, *IFNAR$^{-/-}$RelB$^{-/-}$*, and *IFNAR$^{-/-}$RelB$^{DB/DB}$* mice.

Line graphs of gene expression (CPM) for IFN-independent hyper-expressed upon poly I:C-stimulation (0,1,3, and 8hr) dark blue line (circle) represents WT BMDCs, dark red line (square) represents *RelB$^{-/-}$* BMDCs, dark green line (triangle) represents *RelB*$^{DB/DB}$ BMDCs, light blue line (inverted triangle) represents *IFNAR$^{-/-}$* BMDCs, light red line (diamond) represents *IFNAR$^{-/-}$RelB$^{-/-}$* BMDCs, light green line (circle) represents *IFNAR$^{-/-}$RelB*$^{DB/DB}$ BMDCs.
